# The FIT FIRST 10 dose-response study: evaluation of implementation outcomes

**DOI:** 10.3389/fspor.2025.1504494

**Published:** 2025-02-03

**Authors:** Sofie Koch, Caroline Eckert, Nikos Ntoumanis, Cecilie Thøgersen-Ntoumani, Chiara Cimenti, Malte Nejst Larsen, Peter Krustrup, Lars Breum Skov Christiansen

**Affiliations:** ^1^ Research Unit for Active Living, Department of Sports Science and Clinical Biomechanics, University of Southern Denmark, Odense, Denmark; ^2^ Department of Sports Science and Clinical Biomechanics, Research and Implementation Centre for Human Movement and Learning, University of Southern Denmark, Odense, Denmark; ^3^ Research Unit for Sport and Health Sciences, Department of Sports Sciences and Clinical Biomechanics, University of Southern Denmark, Odense, Denmark; ^4^ Department of Sports Science and Clinical Biomechanics, Danish Centre for Motivation and Behaviour Science (DRIVEN), University of Southern Denmark, Odense, Denmark; ^5^ Department of Sport and Social Sciences, Norwegian School of Sports Sciences, Oslo, Norway; ^6^ School of Sport, Exercise, and Rehabilitation Sciences, University of Birmingham, Birmingham, United Kingdom

**Keywords:** physical activity, implementation, acceptability, appropriateness, feasibility, capability, opportunity, motivation

## Abstract

**Introduction:**

Increasing physical activity (PA) levels among children is critical to mitigate health risks associated with physical inactivity. Schools have been highlighted as ideal setting for promoting PA. However, existing school-based PA programs often face implementation challenges. The FIT FIRST 10 (FF10) multi-sport program has been introduced in Denmark, aiming to increase PA and to enhance children's health, fitness, and well-being. This study evaluates the implementation of the FF10 program for 2nd and 3rd graders.

**Materials and methods:**

The FF10 program was implemented in a 20-week cluster randomized controlled trial across 27 schools. Schools were assigned to a control arm or intervention arms receiving either 3 (full dose) or 1.5 (half dose) FF10 40-min lessons weekly. Teachers received a one-day training session, comprehensive manuals, and necessary equipment. Data were collected from teachers via logbooks documenting implementation fidelity, and an online questionnaire assessing program acceptability, appropriateness, and feasibility, and teachers' capability, opportunity, and motivation for implementing the FF10 program.

**Results:**

A total of 18 intervention schools with 36 classes participated in this study. Program fidelity was high in both intervention groups (2.8 and 2.0 session/week for full and half-dose, respectively). Confidence intervals indicated no differences between the two intervention groups for any outcomes. Teachers (*n* = 32) in both groups rated FF10 moderately acceptable, appropriate, and feasible (3.5–4.0 out of a potential maximum of 5). Both groups exhibited moderate physical and psychological capabilities (3.5–4.0) and high social opportunities (>4.0), but poor motivation scores (<3.5), particularly regarding the perceived benefits and automatization in delivering the FF10 program.

**Conclusion:**

The FF10 program was almost delivered as intended, but time constraints, limited facilities, and modest teacher motivation might have hindered implementation. Reducing the program's dosage did not improve implementation outcomes, emphasizing the need for ongoing support to boost teacher motivation and integration of the program into school timetables.

## Introduction

Increasing children's physical activity (PA) levels is imperative to counteract the health risks associated with rising rates of physical inactivity (1–3). Addressing this widespread issue has led to a strong advocacy for the development and implementation of high-quality school-based initiatives aimed at promoting PA (1). Given that children spend a substantial portion of their waking hours in educational settings, schools represent optimal environments for the broad promotion of PA (4). Despite this, existing school-based PA programs have demonstrated limited effectiveness (5, 6). The perceived shortcomings of these programs are frequently attributed to various implementation challenges, including low program fidelity, low acceptability, poor alignment with the contextual environment, and inadequate access to necessary resources, time, or space (7, 8).

Enhancing the implementation of evidence-based programs and practices is imperative and necessitates behavior change of the implementation agent (9). In the school setting, it is most often the teachers, who need to change their practice. The COM-B model for behavior change offers a robust framework for understanding this process of behavior change (9). According to the COM-B model, behavior results from three critical components (or their interaction): Capability, Opportunity, and Motivation. Capability refers to an individual's physical and psychological capacity to perform a behavior, opportunity encompasses the social or environmental factors that enable or hinder the behavior, and motivation involves the deliberate and automatic processes that drive or withhold the individual to engage in the behavior. In this regard, the implementation agent's perceptions of acceptability, appropriateness, and feasibility of a program is crucial for motivation and for its successful implementation (8, 10). Acceptability pertains to how the program is perceived by the implementation agent, appropriateness relates to the program's fit within the implementation context, and feasibility refers to the practicality of the program's implementation within existing constraints. To succeed with the implementation, it is essential to understand these indicators from multiple perspectives, particularly school staff (11).

Grounded in extensive research and based on recommendations from an international consensus conference in 2016 on children, youth, and PA (12), the FIT FIRST 10 (FF10) program was developed in Denmark in 2018 to enhance physical education (PE) and movement in schools focusing on 10 sports. The program was developed for 2nd and 3rd graders, focusing on a high intensity, inclusive and strength-based approach to teaching (13). The FF10 program aims to be engaging, enjoyable, and inclusive, addressing the diverse needs of students regardless of gender, cultural background, or cognitive abilities. Additionally, the program aligns with the academic standards for PE in primary schools in Denmark.

In 2023 and 2024, the FF10 dose-response study was conducted to investigate the effects of three weekly FF10 lessons compared to half dose, 1.5 weekly lessons, on cardiometabolic and muscular fitness, and well-being (not published yet). A specific priority was to evaluate differences in implementation, which is the primary aim of this study. Specifically, this study aims to: (1) assess the fidelity of FF10 program implementation, (2) evaluate teachers' perceptions regarding the acceptability, appropriateness, and feasibility of the program, and (3) examine teachers' capability, opportunity, and motivation for delivering the FF10 program across the two intervention arms.

## Materials and methods

The Template for Intervention Description and Replication (TIDieR) was used to guide the description of the study (14).

### The FIT FIRST 10 program

The FF10 program comprises 60 lesson plans, developed through collaboration between researchers at the University of Southern Denmark, the Danish Sport Confederation (DIF), Team Denmark (the Danish national elite sports organization), and ten participating sports federations. The program was developed to be accessible to all school staff, regardless of their qualifications, ensuring wide applicability. Each FF10 lesson lasts 40 min and is meticulously designed to elevate children's heart rates through high-intensity activities (average of 75% of maximum heart rate throughout lesson and a minimum of 15 min about 85% of maximum heart rate).

The sports federations involved in developing the FF10 manual include badminton, basketball, flag football (American football), soccer, handball, judo, orienteering, rugby, taekwondo, and volleyball. The FF10 program can be incorporated into the PE curriculum or utilized as extracurricular PA.

To support the implementation of the FF10 program, participating teachers from the intervention schools undertook a one-day face-to-face training session designed to impact the necessary knowledge and skills for effective implementation of the FF10 program prior to the commencement of the program. The courses were run by members of the FIT FIRST FOR ALL project group, often companied by representatives from the sports federations. The courses included a 1 h theoretical presentation, giving the teachers an introduction to the evidence underpinning the FIT FIRST program, as well as the three principles on which the program is based: high intensity, fun, and inclusion of all students. Following the presentation, two practical sessions were conducted in a sports hall. In the first session, teachers had the opportunity to engage directly with the three principles of the program. In the second session, they were presented with a complete lesion plan from two of the participating sports. This provided insight into the structure of the lessons and showcased the variety of activities across different sports.

Throughout the sessions, the FIT FIRST instructors psed reflective questions to encourage teachers to consider how the concept could be adapted to specific contexts, such as the available facilities. Teachers were also given the opportunity to ask clarifying questions during the sessions. The course concluded with a discussion on how the participating schools could ensure the implementation of the 1.5 or three weekly FF10 lessons.

The course was designed to accommodate both physical education (PE) and non-PE teachers, ensuring that all participants, regardless of their professional background, were prepared to implement FF10 afterward. The theoretical component was perceived by many PE teachers as content they were already familiar with, while a significant number of non-PE teachers found this part particularly beneficial. Similarly, the practical component offered distinct takeaways for the two groups. PE teachers expressed gaining new inspiration for their teaching, whereas non-PE teachers emphasized that the course provided them with a clearer understanding and skills on how to work with FF10—a skill that PE teachers generally felt confident about even before attending the course.

In this way, the course yielded different benefits depending on the teachers' backgrounds but was particularly structured to support those less accustomed to incorporating movement into their teaching practices.

The courses were conducted either at one of the participating schools or in sports halls across the country, where nearby schools participated. During the courses, participants were also provided with comprehensive manuals containing lesson plans for all ten sports included in the program. Additionally, each intervention school received equipment (e.g., balls, cones, scrimmage vests) required for execution of some FF10 lessons.

### Study population and data collection

The FF10 dose-response study was a 20-week cluster randomized controlled trial with a 1:1:1 allocation ratio. All schools in Denmark, both public and private, were invited to participate in the study. The recruitment process involved disseminating invitations online via LinkedIn and the DIF's homepage, as well as through direct email outreach. The email invitations were initially sent to schools in the region of Southern Denmark and the Capital region, followed by other regions, until the desired number of participants was reached. After sending the emails, a personal follow-up call was made to each school to inquire whether they were interested in participating in the study. Recruitment took place over a 12-month period, from October 2022 to December 2023. A total of 27 schools were recruited, with 12 schools participating in the study from January–June 2023, and 15 schools participating in the study from January–June 2024.

The schools were randomly assigned to one of three arms: a control arm, an intervention half-dose arm, which received 1.5 FF10 lessons per week, and an intervention full-dose arm, which took part in three FF10 lessons per week. In the intervention group receiving 1.5 FF10 lessons per week, they were asked to deliver one lesson one week and two lessons the next week, giving an average of 1.5 lessons across the intervention period. The FF10 lessons were conducted during regular school hours and were a part of PE or replaced other subjects/classes during the week.

The group of schools participating in 2023 was allocated as follows: four schools in the control arm, five schools in the half-dose arm, and three schools in the full-dose arm. In 2024, the distribution was as follows: five schools were assigned to the control arm, four schools to the half-dose arm, and six schools to the full-dose arm, giving a total of nine schools in each arm and a total of 1,358 children at baseline. Due to two weeks of public holidays during the intervention period and scheduling of data collection, the schools participating in 2023 had an intervention period lasting 17 weeks, while the schools participating in 2024 had a program lasting 18 weeks. This study will therefore be based on the reduced number of weeks rather than the 20 weeks as intended. Data from the intervention schools were the focus of this study, which was collected at eight schools in 2023 (three full dose and five half-dose schools) and ten schools in 2024 (six full dose and four half-dose schools). Additionally, as the program was designed to promote PA in schools and because the schools were asked to deliver either 1.5 or three weekly FF10 lessons, both PE and non-PE teachers were involved in the delivery of the program, as schools in Denmark only have two PE lessons per week.

### Logbooks

Teachers responsible for implementing the FF10 program in each class were requested to complete a weekly logbook documenting the FF10 activities conducted for each participating class. Informed by Proctor et al. (8), the logbook comprised questions to assess program fidelity. Specifically, the teachers were asked to record: (1) the number of FF10 lessons delivered, (2) the sports activities covered in each lesson, and (3) the total time spent on FF10 activities each week. For question 2, teachers selected the type of sport from a predefined list, with an additional option to specify “other” if activities deviated from those outlined in the FF10 manual. Logbook data were collected from January to June 2023 at eight schools and from January to June 2024 at the remaining ten schools.

### Questionnaire

Following the intervention period, participating teachers (*N* = 40) received a link to an online questionnaire. This questionnaire aimed to gather data on various aspects of the FF10 program, including its acceptability, appropriateness, feasibility, as well as teachers' capabilities, opportunities, and motivation related to its implementation. To assess acceptability, appropriateness, and feasibility, the questionnaire incorporated the validated Acceptability of Intervention Measure (AIM), Intervention Appropriateness Measure (IAM), and Feasibility of Intervention Measure (FIM) developed by Weiner et al. (10), which consist of 12 questions, four questions related to each of the three measures (AIM, IAM, FIM). In this study, six of the questions were included, two for each of the three measures (e.g., “FIT FIRST is appealing to me”, “FIT FIRST seems fitting”).

Additionally, questions addressing capabilities, opportunities, and motivation were adapted from the questionnaire developed by Verdenschot et al. (15), centering on the COM-B model for behavior change (see Supplementary Materials S1) (9). A total of 18 questions related to the COM-B framework were included in the questionnaire. Four questions assessed capability (e.g., “I know how to deliver FIT FIRST”), eight questions examined opportunity (e.g., “I have enough time to plan the delivery of FIT FIRST”), and six questions evaluated motivation (e.g., “I am motivated to deliver FIT FIRST 10”). A 5-point Likert scale was employed for both the AIM, IAM, and FIM questions, and questions related to the COM-B model: (1) “Strongly disagree”, (2) “Disagree” (3) “Neither agree or disagree”, (4) “Agree”, and (5) “Strongly agree”). Both the AIM, IAM, and FIM and the COM-B questions were translated to Danish, following the guidelines for cross-cultural adaptations of self-report measures by Beaton et al. (16). The questionnaire also included items on the teachers' PE backgrounds (e.g., “are you a trained physical education teacher?”) and provided an open field for additional comments about the program. The questionnaire data were collected between May and June 2023 from eight intervention schools, and between May and June 2024 from the remaining ten intervention schools.

### Data analysis

Data were analyzed using STATA 18, generating descriptive statistics. The analysis of the AIM, IAM, and FIM followed the scoring guidance provided by Weiner et al. (10). Due to the absence of specific cut-points for these tools, a scoring system from other studies was applied (17, 18). Scores below 3.5 were considered poor, scores between 3.5 and 4.0 were deemed moderate, and scores above 4.0 were regarded as high in terms of acceptability, appropriateness, and feasibility (17, 18). The same scoring system was used for the questions related to the COM-B model. Due to the relatively small sample size, confidence intervals were used for both the AIM, IAM, and FIM questions, and for the questions related to COM-B to examine potential differences between the two intervention arms.

### Ethical considerations

Logbooks and questionnaires were anonymized, ensuring that no personally identifiable information, such as names and civil registration data was collected. A passively informed consent procedure was used, wherein teachers were automatically included in the study unless they actively chose to withdraw consent. This procedure has been found to be ethically appropriate in low-risk research and is in accordance with Danish regulations. To opt out, teachers were required to notify the research team using the contact information provided with the questionnaire invitation. The study was given a waiver by the Health Research Ethics Committee of the Region of Southern Denmark (S-20222000–108) and approved by the legal service department at University of Southern Denmark (11.806) and conducted in accordance with the World Medical Association Declaration of Helsinki.

## Results

All intervention schools (*N* = 18) provided data for this study. A total of 42 classes were invited, of which teachers from 38 classes completed the logbooks. In the full dose arm, 10.5% of the classes implemented FF10 in addition to PE, whereas 45.8% of the classes in the half-dose arm did so. The remaining classes integrated FF10 within their PE lessons (Table 1).

**Table 1:** Characteristics of the two groups providing data for the study.

	Full dose (3 times/week)	Half-dose (1.5 times/week)
Schools, *n*	9	9
Classes, *n*	18	20
Classes with FF10 in addition to PE,% (*n*)	10.5 (2)	45.8 (11)
Grades	2nd, 3rd	2nd, 3rd
Students, *n*	337	584
PE teachers,% (*n*)	38.5 (5)	47.4 (9)

Table 2 outlines fidelity related to program requirements. Neither intervention arm achieved an average of 17–18 weeks of intervention, but some classes in both arms managed to deliver the FF10 program for the full intervention period. Classes in the full dose arm conducted an average of 2.6 weekly FF10 lessons, while the half-dose arm conducted an average of 1.5 FF10 lessons per weekly throughout the 17–18-week intervention period, of which 2.1 and 1.4 lessons, respectively, were based on FF10 materials. Looking at the average for the weeks where the schools delivered the FF10 sessions, schools in the full dose arm had an average of 2.8 weekly FF10 lessons, while the half-dose arm delivered 2.0 weekly FF10 lessons. The full dose arm delivered on average 2.2 weekly lessons based on FF10 materials, whereas the half-dose arm delivered an average of 1.8 weekly lessons based on FF10 materials throughout the weeks where they delivered the program (16.4 and 13.2, respectively). Thus, 0.8 weekly lessons in the full dose arm and 0.2 weekly lessons in the half-dose arm were based on activities other than the FF10 program (e.g., rounders, going for a walk). Teachers in the full-dose arm delivered an average of 118.7 min weekly, while 56.2 weekly minutes were delivered in the half-dose arm throughout the 17–18-week intervention period. Looking at the weeks where they delivered the FF10 program, the full dose arm delivered an average of 123.2 min weekly and the half-dose arm delivered 79.5 weekly minutes of FF10.

**Table 2:** Descriptive statistics on fidelity across the two intervention arms for the weeks where they were delivering the FF10 program.

Outcomes	Full-dose	Half-dose
Weeks with FF10 (Mean, SD, range)	16.4 [2.2, (13–18)]	13.2 [5.6, (3–18)]
FF10 lessons per week (Mean, SD, range)	2.8 [0.13, (2.7–3.0)]	2.0 [0.63 (0.7–3.0)]
FF10 lessons per week with FF10 content (Mean, SD)	2.2 (0.69)	1.8 (0.78)
Minutes per week^a^ (Mean, SD)	123.2 (14.5)	79.5 (24.7)

^a^ In weeks where FF10 lessons were logged. Average for 17–18 weeks of intervention was 118.7 min and 56.2 min, respectively.

Due to the different proportion of PE teachers between the two intervention arms, additional analyses were conducted comparing PE teachers to non-PE teachers to examine potential differences between these two teacher groups.

The results of the questionnaire related to acceptability, appropriateness and feasibility are presented in Table 3. Scores between 3.5 and 4.0 imply that the teachers in average found FF10 moderately acceptable, appropriate, and feasible and the confidence intervals indicated no differences between intervention arms or teacher groups.

**Table 3:** Results of the measures of acceptability, appropriateness, and feasibility across the two intervention arms and across PE teachers and non-PE teachers.

	Full dose	Half dose	PE teachers	Non-PE teachers
	[Mean (95% CI)]	[Mean (95% CI)]	[Mean (95% CI)]	[Mean (95% CI)]
	*N* = 13	*N* = 19	*N* = 14	*N* = 18
AIM^a^	4.0 (3.54; 4.54)	3.7 (3.24; 4.13)	4.0 (3.43; 4.57)	3.7 (3.28; 4.10)
IAM^a^	3.7 (3.12; 4.18)	3.6 (3.19; 4.02)	3.6 (3.09; 4.13)	3.6 (3.22; 4.05)
FIM^a^	3.8 (3.34; 4.28)	3.7 (3.30; 4.12)	3.8 (3.36; 4.28)	3.7 (3.28; 4.11)

^a^ AIM, acceptability of intervention measure; IAM, intervention appropriateness measure; FIM, feasibility of intervention measure. Scores out of 5, with 5 indicating best possible score Scores <3.5 were deemed poor, 3.5–4.0 were deemed moderate, and scores of >4.0 were deemed high acceptability, appropriateness and feasibility (17).

The results from the COM-B questions are presented in Table 4. Results showed that the full dose arm generally exhibited high scores in both physical and psychological capabilities necessary for implementing the FF10 program. However, a moderate score was observed for the question concerning the ability to deliver the FF10 program when facing barriers. In contrast, the half-dose arm showed moderate scores ranging from 3.6–3.8 for most questions related to physical and psychological capabilities, except for a high score on the question regarding their knowledge of how to deliver the program. Based on the confidence intervals there seemed to be no difference between the two intervention arms in terms of physical or psychological capabilities.

**Table 4:** Results of the COM-B measures of capability, opportunity, and motivation across the two intervention arms and across PE teachers vs. not PE teachers.

	Full-dose group	Half-dose group	PE teachers	Non-PE teachers
	(*n* = 13)	(*n* = 19)	(*n* = 14)	(*n* = 18)
	[mean (95% CI)]	[mean (95% CI)]	[mean (95% CI)]	[mean (95% CI)]
**Physical capability subscale score**	4.3 (3.82; 4.88)	3.8 (3.28; 4.24)	4.0 (3.42; 4.58)	4.0 (3.51; 4.49)
I have the physical fitness to deliver FIT FIRST (Physical capability^a^)	4.3 (3.74; 4.88)	3.7 (3.18; 4.29)	3.9 (3.23; 4.63)	4.0 (3.48; 4.51)
I have the physical skills to deliver FIT FIRST (Physical capability^a^)	4.4 (3.86; 4.91)	3.8 (3.35; 4.23)	4.1 (3.59; 4.55)	4.0 (3.49; 4.51)
**Psychological capability subscale score**	4.0 (3.51; 4.49)	3.9 (3.55; 4.19)	4.1 (3.73; 4.86)	3.8 (3.40; 4.15)
I know how to deliver FIT FIRST (Psychological capability^a^)	4.2 (3.79; 4.67)	4.3 (3.95; 4.58)	4.5 (4.20; 4.80)	4.1 (3.69; 4.42)
I can deliver FIT FIRST even when barriers emerge (Psychological capability^a^)	3.8 (3.16; 4.38)	3.5 (3.01; 3.94)	3.7 (3.10; 4.33)	3.5 (3.04; 3.96)
**Physical opportunity subscale score**	3.5 (3.04; 3.91)	3.6 (3.32; 3.97)	3.6 (3.15; 4.14)	3.5 (3.25; 3.79)
My school has the physical facilities to deliver FIT FIRST (Physical opportunity^a^)	4.2 (3.56; 4.75)	3.9 (3.54; 4.36)	3.9 (3.35; 4.50)	4.1 (3.70; 4.53)
My school has the equipment to deliver FIT FIRST (Physical opportunity^a^)	3.2 (2.53; 3.94)	3.6 (3.21; 3.95)	3.6 (2.94; 4.20)	3.3 (2.92; 3.75)
I have enough time to plan FIT FIRST (Physical opportunity^a^)	3.0 (2.45; 3.55)	3.4 (2.97; 3.77)	3.4 (2.82; 3.89)	3.1 (2.70; 3.53)
I have enough time to deliver FIT FIRST (Physical opportunity^a^)	3.5 (3.06; 3.86)	3.4 (2.88; 3.96)	3.4 (2.72; 4.13)	3.4 (3.09; 3.79)
I found the FIT FIRST resources easy to implement in my school (Physical opportunity^a^)	3.5 (3.07; 4.01)	3.9 (3.50; 4.28)	3.9 (3.35; 4.50)	3.6 (3.31; 3.91)
**Social opportunity subscale score**	4.2 (3.85; 4.56)	4.2 (3.90; 4.41)	4.2 (3.91; 4.47)	4.2 (3.87; 4.46)
I have the necessary support from the school executives to deliver FIT FIRST (Social opportunity^a^)	4.2 (3.73; 4.73)	4.2 (3.87; 4.55)	4.3 (3.93; 4.64)	4.2 (3.74; 4.59)
I have the necessary support from my colleagues to deliver FIT FIRST (Social opportunity^a^)	4.2 (3.67; 4.64)	4.2 (3.76; 4.56)	4.0 (3.49; 4.51)	4.3 (3.90; 4.65)
I have the necessary support from parents and guardians to deliver FIT FIRST (Social opportunity^a^)	4.2 (3.87; 4.59)	4.1 (3.83; 4.38)	4.3 (4.02; 4.56)	4.1 (3.74; 4.37)
**Reflective motivation subscale score**	3.6 (3.21; 3.94)	3.6 (3.26; 3.85)	3.5 (3.22; 3.86)	3.6 (3.26; 3.91)
I can see the benefits of delivering FIT FIRST (Reflective motivation^a^)	3.0 (2.45; 3.55)	2.9 (2.58; 3.21)	2.7 (2.24; 3.19)	3.1 (2.77; 3.45)
I am planning to deliver FIT FIRST (Reflective motivation^a^)	3.7 (3.24; 4.15)	3.6 (3.09; 4.07)	3.6 (3.03; 4.11)	3.7 (3.22; 4.12)
I am motivated to deliver FIT FIRST (Reflective motivation^a^)	3.8 (3.21; 4.33)	3.9 (3.50; 4.28)	3.9 (3.41; 4.30)	3.8 (3.37; 4.29)
My students are motivated to participate in FIT FIRST (Reflective motivation^a^)	3.8 (3.36; 4.33)	3.8 (3.41; 4.28)	4.0 (3.55; 4.45)	3.7 (3.28; 4.17)
**Automatic motivation subscale score**	3.5 (3.04; 3.96)	3.5 (2.91; 4.03)	3.5 (2.82; 4.11)	3.5 (3.03; 3.97)
I enjoy delivering FIT FIRST (Automatic motivation^a^)	3.6 (3.10; 4.14)	3.6 (3.05; 4.22)	3.5 (2.83; 4.17)	3.7 (3.22; 4.23)
Delivering FIT FIRST is part of my routine (Automatic motivation^a^)	3.4 (2.92; 3.85)	3.3 (2.74; 3.90)	3.4 (2.76; 4.10)	3.3 (2.80; 3.75)

^a^ Scores out of 5, with 5 indicating best possible score. Scores are calculated as means for each intervention arm and teacher group. Scores < 3.5 were deemed poor, 3.5– 4.0 were deemed moderate, and scores of > 4.0 were deemed high acceptability, appropriateness and feasibility (17).

For opportunity, the confidence intervals indicated no differences between the two intervention arms either. The full dose arm showed a high score on the question related to having the physical facilities to deliver FF10, whereas the half-dose arm showed a moderate score on this question. The full dose arm showed a poor score on the question related to having the necessary equipment for implementing FF10, in which the half-dose arm showed a moderate score. Both intervention arms showed poor scores for the questions related to having enough time to plan and deliver the program, moderate scores on the question related to finding the FF10 resources (e.g., the manual) easy to implement in their schools, and high scores for all the questions related to social opportunities to FF10 implementation. Results from the confidence intervals indicated no difference between the two intervention arms for any of the questions related to either physical or social opportunity.

For motivation, both intervention arms showed similar scores, and confidence intervals indicated no differences for any of the questions. Both intervention arms showed poor scores on the question related to seeing the benefits of implementing FF10 and the question on whether delivering FF10 had become part of their routine. On all other questions related to motivation, both intervention arms showed moderate scores.

Due to overlap in confidence intervals, there seems to be no difference between the two teacher groups with respect to acceptability, appropriateness, feasibility, or any of the components related to the COM-B model.

Results from the open comment field showed that most teachers found the concept valuable, but time-consuming both in terms of planning and delivering the program. Especially, integration of FF10 into preplanned timetables was identified as a challenge in the open comment field. Moreover, some teachers indicated that while the program was suitable for PE teachers, it might be challenging to implement for those who are not PE teachers. Furthermore, getting access to appropriate facilities (e.g., gym hall) was also mentioned as a challenge for the implementation of FF10.

## Discussion

The present study aimed to evaluate the fidelity, the teachers' perceived acceptability, appropriateness, feasibility, as well as their capability, opportunity, and motivation related to the implementation of FIT FIRST 10 over a 20-week period in 2nd and 3rd grade, delivered either 1.5 times per week or 3 times per week.

### Fidelity

Overall, the study found that the full-dose arm almost delivered the targeted 17–18 weeks of intervention (16.4 weeks) on average, whereas the half-dose arm delivered an average of 13.2 weeks of intervention. Several classes in the half-dose arm completed only a few weeks of the program. Responses from the open comment field suggest that integrating FF10 lessons posed challenges for some schools, potentially contributing to incomplete program implementation. Schools had to adjust their timetables to accommodate FF10, often requiring other subjects to make room if the program was not delivered during PE.

Both intervention arms demonstrated high fidelity in terms of the weekly frequency of FF10 lessons delivered. However, adherence to the prescribed FF10 content varied, with the half-dose arm delivering more lessons aligned with the FF10 content compared to the full dose arm. Schools, as busy environments focused on teaching and learning, often struggle to implement additional PA programs (19–21). This challenge may partly explain the variance between the two intervention arms, particularly as the full dose arm was required to deliver twice the amount of PA as the half-dose arm.

When examining dose-response between the intervention arms, several key points emerge. The half-dose arm often used FF10 as a supplement to PE, which may have affected how it was integrated into curriculum. Interestingly, a larger number of classes in the half-dose arm failed to complete the full 20-week intervention, raising the question whether this was coincidental or indicative of broader implementation challenges. Especially, when implementing the concept out of PE, as many of classes in the half-dose group did. Although the program was designed to promote PA across the school daya and be applicable in any subject, these results suggest that it may fit best within PE, likely due to the practical constraints of school schedules that make it difficult for most schools to allocate time outside of existing PE lessons.

Additionally, the half-dose arm appeared to deliver more sessions per week, possible compensating for missed weeks. This aligns with the observation that some weeks were not completed as planned. In contrast, the full dose arm nearly met their target of three lessons per week. When looking at the total time spent on the program, the half-dose arm seems to exceed expectations when lessons occurred regularly, while the full dose arm adhered to the intended schedule during active weeks. If calculated over the intended 20-week period, the half-dose arm may have effectively delivered the equivalent of half the intended dose.

### Acceptability, appropriateness, and feasibility

The findings of moderate acceptability, appropriateness, and feasibility (scores between 3.5–4.0) are consistent with a study by Nathan et al. (17), which assessed the impact of a multi-strategy PA program in schools. Additionally, Mclaughlin et al. (18) examined the acceptability, appropriateness, and feasibility of a whole-school secondary PA program in a scale-up trial, revealing that teachers found the program highly acceptable, appropriate, and feasible. The confidence intervals indicated no differences between the two intervention groups in terms of acceptability, appropriateness, or feasibility. This finding suggests that there may be no difference between the full dose and the half-dose arm from an implementation perspective regarding teachers' perceptions of the program's acceptability, appropriateness, and feasibility. However, the moderate scores suggest that there is room for improvement of the program for both groups in terms of making it more acceptable, appropriate, and feasible to implement within a school context.

Previous studies have shown the value of implementation strategies on implementation outcomes, as they constitute the “how to” component to changing practice (17, 18, 22). In this study, three implementation strategies were applied for the teachers: a one-day course, a manual with lesson plans, and a pack of equipment to support the implementation. Previous studies have applied strategies such as using in-school champions to promote the program, centralizing technical assistance, providing ongoing consultations, and capturing and sharing local knowledge (18, 19, 23, 24). In this study, teachers would probably have benefitted from ongoing support for the FF10 delivery, such as for assistance in integrating FF10 into timetables and how to use the existing facilities at the schools, which might not always be the gym hall.

No differences were observed between PE and non-PE teachers on their perception of acceptability, appropriateness, and feasibility. However, responses from the open comment field revealed that the concept was more suitable for PE teachers and could be challenging to implement for a non-PE teacher. This challenge may call for differentiated training and support provided prior to program implementation. PE teachers are typically accustomed to working with this type of program material (i.e., the manual with lesson plans) and teaching in a PE setting, whereas non-PE teachers may find it more difficult to translate the knowledge from the program materials onto practice, which aligns with previous studies (25, 26).

### Capabilities, opportunities, and motivation

Confidence intervals indicated no differences between the two intervention arms regarding any of the COM-B model components. However, the results related to capabilities revealed that the full dose arm scored high on physical capabilities, such as possessing the physical fitness and competencies necessary for FF10 delivery, compared to the half-dose arm, which showed moderate scores. This difference suggests that teachers in the full dose arm felt more prepared and confident in their ability to deliver the FF10 program, aligning with research indicating that sufficient training and support can enhance teachers' self-efficacy and competence (27, 28).

In terms of psychological capabilities, both intervention arms reported high scores related to knowledge needed for FF10 delivery and moderate scores concerning their ability to manage barriers. Overall, both intervention arms demonstrated moderate to high scores across all capability-related questions, indicating a general sense of competence in implementing the program.

Regarding physical opportunities, the full dose arm reported a high score on having access to the necessary facilities for FF10 delivery, whereas the half-dose arm reported a moderate score. Facilities are a known barrier to PA implementation in schools, suggesting that the study findings are positive since integrating additional PA requires logistical adjustments to accommodate available facilities, which are often reserved for PE classes (19, 29). The full and half-dose arm scored poor and moderate, respectively, on having the necessary equipment for FF10 delivery, based on the scoring system used in previous studies (17, 18). The study did only provide partial equipment support, which seemed to be a particular challenge for the full dose arm, that were expected to deliver twice the amount of FF10 lessons. Time constraints were also noted as a barrier, consistent with previous research highlighting time as a major obstacle to PA implementation (19, 29). As FF10 was an add-on to the existing curriculum (despite PE curriculum), teachers had to balance it with their regular teaching responsibilities.

For social opportunities, both intervention arms scored high, indicating strong support from school management, colleagues, and parent or guardians, which aligns with the importance of a supportive school culture for successful program adoption and implementation (30–32).

In terms of reflective motivation, both intervention arms exhibited modest scores regarding the perceived benefits of FF10 implementation. These modest scores may reflect a lack of perceived value or understanding of the program's benefits compared to regular PE or personal preferences. Moreover, both intervention arms also showed modest scores for automatic motivation and the question related to FF10 being part of their routine. This suggest that the program has not yet become part of teachers' routine and may require additional time to integrate fully and recognize its benefits. Given that teacher motivation is crucial for the success of PA implementation, addressing these motivational challenges through increased support could be vital for sustaining the implementation of FF10 (9).

Confidence intervals indicated no differences between PE and non-PE teachers in terms of motivation. However, PE teachers reported moderate availability of necessary equipment for FF10, while non-PE teachers reported poorer access. This discrepancy may be attributed to PE teachers' greater familiarity with school resources. Consequently, non-PE teachers might benefit from additional support, such as consultations during the pre-implementation and adoption phase of FF10.

### Strengths and limitations

The FF10 program is grounded in evidence-based practice, including a wide range of sports and activities designed to increase heart rate through high-intensity activities that are engaging for children (13). The inclusion of both PE and non-PE teachers is a strength, providing valuable insights, which could be used to inform future roll-out of the program. The study also has some limitations. First, the generalizability of the findings may be restricted due to the relatively small sample size. While the FF10 program is grounded in evidence-based practice, incorporating diverse high-intensity activities designed to engage children and increase heart rates (13), the outcomes may not fully translate to broader or more varied settings.

Second, the methods employed for data collection and analysis may have introduced potential biases and may not have accounted for all confounding variables, which could influence the internal validity of the study. For instance, the reliance on self-reported data through logbooks and questionnaires, although supported by previous studies (33, 34), makes the findings susceptible to biases such as recall errors and social desirability, potentially leading to inflated estimates of physical activity levels and program adherence.

Third, teacher motivation, which was not assessed in this study, represents another limitation. Ample evidence highlights the role of teacher motivation in shaping their emotional, cognitive, and behavioral investment in their work, which could also extend to their willingness to implement and sustain a new physical activity program (35).

Additionally, while the study provides valuable insights, the interpretation of the results would benefit from additional studies that replicate or compare findings across similar interventions. Such research would enhance the robustness of the conclusions and support a broader application of the program across different contexts.

Finally, a longer follow-up period in future studies would be beneficial, particularly for evaluating the COM-B variables. This would allow for a more thorough examination of real behavior change over time and provide insights into the sustainability of the intervention's effects (9).

## Conclusion

This study indicated that the FF10 multisport program generally achieved high implementation fidelity. While the program's intended frequency was met, variations in adherence to content were observed between the two intervention arms. Teachers perceived the program as moderately acceptable, appropriate, and feasible, with no observed differences between the two groups based on confidence intervals. Nonetheless, opportunities remain to further adapt the program to the school context. No differences seemed to be present between teacher's perceptions of their capabilities, opportunities, and motivation for FF10 implementation. However, time constraints and inadequate facilities were identified as key challenges. Notable, motivation levels, particularly regarding the perceived benefits of FF10, were modest across both intervention arms and teacher groups, indicating the need for enhanced support to boost teacher engagement. Thus, these findings suggest that reducing the program dosage does not improve implementation outcomes. Future research should investigate the impact of multi-faceted implementation strategies to strengthen teacher motivation and program integration.

## Data Availability

The raw data supporting the conclusions of this article will be made available by the authors, without undue reservation.
