# Supplementary material for: The FIT FIRST 10 dose-response study: evaluation of implementation outcomes
Source: Front Sports Act Living. 2025 Feb 3;7:1504494. doi: 10.3389/fspor.2025.1504494 (PMC11830660; doi:10.3389/fspor.2025.1504494)
Supplement: Supplementary file 1 [file Table1.docx]

Supplementary Material

# Questions used to cover the determinants of the COM-B model for behavior change

Danish version

| **Capability** | |
| --- | --- |
| **Psychological domain** | |
| Jeg har den fysiske form (f.eks. fleksibilitet, kondition og muskelstyrke) til at udføre FIT FIRST 10  Jeg har de fysiske evner (f.eks. evnen til at demonstrere/vise aktiviteterne) til at udføre FIT FIRST 10 | Meget uenig  Uenig  Neutral  Enig  Meget enig |
| **Physical domain** | |
| Jeg ved, hvordan jeg skal udføre FIT FIRST 10  Jeg kan udføre FIT FIRST 10 selv når udefrakommende barrierer opstår (f.eks. mangel på tid) | Meget uenig  Uenig  Neutral  Enig  Meget enig |
| **Opportunity** | |
| **Physical domain** | |
| Min skole har de fysiske faciliteter (f.eks. adgang til gymnastiksal eller passende områder indendørs eller udendørs) til at kunne udføre FIT FIRST 10  Min skole har udstyret (f.eks. træningselastikker, bolde, aktivitetsskemaer) til at udføre FIT FIRST 10  Jeg har tid nok til at planlægge udførelsen af FIT FIRST 10  Jeg har tid til at udføre FIT FIRST 10  Jeg har let ved at anvende ressourcerne (aktiviteter og materialer) fra FIT FIRST 10 på min skole | Meget uenig  Uenig  Neutral  Enig  Meget enig |
| **Social domain** | |
| Jeg har den nødvendige opbakning fra skolens ledelse (f.eks. skoleinspektør eller afdelingsleder) til at udføre FIT FIRST 10  Jeg har den nødvendige opbakning fra mine kollegaer til at udføre FIT FIRST 10  Jeg har den nødvendige opbakning fra forældre og værger til at udføre FIT FIRST 10 | Meget uenig  Uenig  Neutral  Enig  Meget enig |
| **Motivation** | |
| **Reflective domain** | |
| Jeg kan se fordelene (f.eks. forbedringer i elevernes opførsel i klasseværelset)  Jeg har planer om at forsætte med FIT FIRST 10, hvis jeg får muligheden for det  Jeg er motiveret til at udføre FIT FIRST 10  Mine elever er motiveret til at deltage i FIT FIRST 10 | Meget uenig  Uenig  Neutral  Enig  Meget enig |
| **Automatic domain** | |
| Jeg nyder at udføre FIT FIRST 10  At udføre FIT FIRST 10 er en del af min rutine | Meget uenig  Uenig  Neutral  Enig  Meget enig |

**English version**

| **Capability** | |
| --- | --- |
| **Psychological domain** | |
| I have the physical fitness (i.e., flexibility, aerobic and muscular fitness) to deliver FIT FIRST 10  I have the physical skills (e.g., I can demonstrate the activities) to deliver FIT FIRST 10 | Strongly disagree  Disagree  Neutral  Agree  Strongly agree |
| **Physical domain** | |
| I know how to deliver FIT FIRST 10  I can deliver FIT FIRST 10 even when barriers emerge (e.g., lack of time) | Strongly disagree  Disagree  Neutral  Agree  Strongly agree |
| **Opportunity** | |
| **Physical domain** | |
| My school has the physical facilities (e.g., access to a gym or appropriate indoor or outdoor space) to deliver FIT FIRST 10  My school has the equipment (e.g., resistance bands, balls, activity cards) to deliver FIT FIRST 10  I have enough time to plan the delivery of FIT FIRST 10  I have enough time to deliver FIT FIRST 10  I found FIT FIRST 10 resources easy to implement in my school | Strongly disagree  Disagree  Neutral  Agree  Strongly agree |
| **Social domain** | |
| I have the necessary support from school executives (e.g., principal or Head of Department) to deliver FIT FIRST 10  I have the necessary support from my colleagues to deliver FIT FIRST 10  I have the necessary support from parents and guardians to deliver FIT FIRST 10 | Strongly disagree  Disagree  Neutral  Agree  Strongly agree |
| **Motivation** | |
| **Reflective domain** | |
| I can see the benefits (e.g., improvements in students’ classroom behaviour) of delivering FIT FIRST 10  I am planning to deliver FIT FIRST 10  I am motivated to deliver FIT FIRST 10  My students are motivated to participate in FIT FIRST 10 | Strongly disagree  Disagree  Neutral  Agree  Strongly agree |
| **Automatic domain** | |
| I enjoy delivering FIT FIRST 10  Delivering FIT FIRST 10 is part of my routine | Strongly disagree  Disagree  Neutral  Agree  Strongly agree |

**
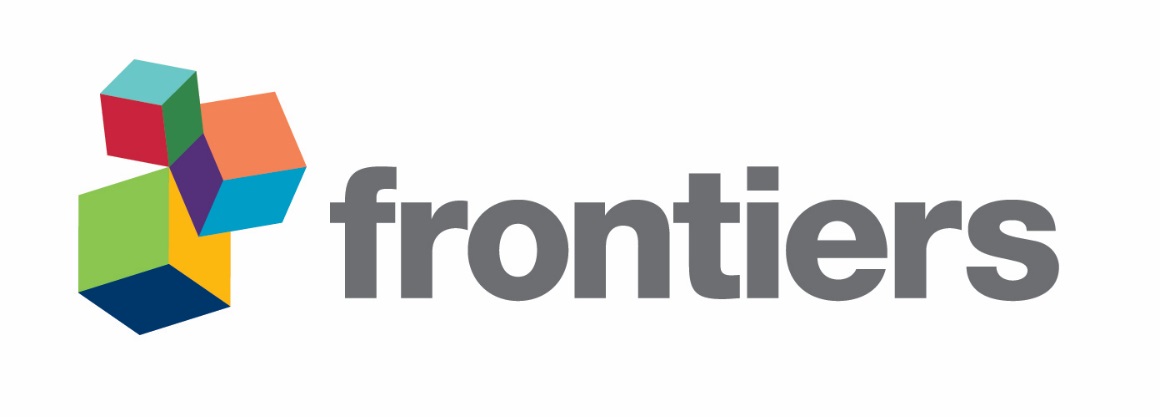
**
